# The Influence of Uræmic and Alcoholic Poisoning on Testamentary Capacity

**Published:** 1873-05

**Authors:** Stephen Rogers


*The Influence of Uræmic and Alcoholic Poisoning on Testamentary Capacity.* Read before the New York Medico-Legal Society, by STEPHEN ROGERS, M.D.

Near the close of the year 1869, E. I. C. was quietly, if not clandestinely, married to a man whom, report says, her education, religious and secular, would have, in the ordinary course of events, led her to shun, rather than to love.

A key to the secret of this untoward occurrence is furnished by accumulated evidence, to the effect that she was an incorrigible and hopeless inebriate. Though trained, and fairly educated, in one of the best schools for girls, this vice of drunkenness seized her soon after her return home from school, and, as a consequence, she soon became the cause of constant and harassing care to, and her habits were the bane of the last days of her aged and widowed mother, who died about the date above given, leaving this only child sole heir to a considerable property.

About the time of the mother's death, this rich and dissolute girl was induced to marry, under the circumstances already stated. Rumor attributes the marriage to a necessity arising from antecedent illicit familiarity between the parties, favored by the intemperate habits of the girl. However this may be, there is no question that they were married, and the following unusual history commenced: Not long after her marriage, it appears in evidence, she became the victim of attacks which, from the general description given of them by various witnesses, would appear to have been of an hysterical character, more or less modified by her intemperate habits, for these she continued to practice after her marriage with even less restraint than before.

During these attacks she screamed, talked incoherently, and threw herself about so violently as to require control, and she is said to have recovered from them after a few minutes, or at most a few hours, with no perceptible impairment of her intelligence. It also became apparent, not long after her marriage, that she was pregnant, to which fact we may justly attribute a portion of the general and violent nervous perturbation which she at this time suffered. This perturbation was manifested, as before stated, in an especial manner by the attacks, or spells, or fits, or paroxysms, as they are variously named by the witnesses, whose principal features I have already mentioned; and, except these, no alarming symptom occurred to her till about eight months after marriage, though the evidence is conclusive that she had, before this date, suffered many of the aforementioned attacks, and had more than once been on the verge of true delirium tremens. Either from the fear that some of those paroxysms might terminate fatally, or that her uncontrolled intemperance might induce disease which would suddenly terminate her life, or from some other fear or motive, her husband and her father-in-law, who were associated in business, went, unknown to her, some months after the marriage, to an attorney, and requested him to draw up a will for her, at the same time giving him instructions as to its provisions. The will was accordingly prepared, and in such terms as to be almost exclusively in the interest of the two parties who had ordered it drawn. They took it, carried it home, and laid it away in a trunk, as the evidence shows. There is no evidence furnished to prove that the alleged testatrix ever read, or heard this paper read, except the declarations of the parties who caused the will to be drawn. It is from them alone that we hear that she even knew of the existence of this paper till almost the hour of her death. Her husband, who, by its terms, was sole heir to her estate, alone says she approved it after reading it, and still months passed, and it was allowed to lie away in his trunk unexecuted and unwitnessed.

Thus stood the affairs of this case when, on a Monday, this alleged testatrix exhibited signs of the approach of one of her accustomed ill-turns, which, as already said, the evidence all goes to prove, were the result of alcoholic excesses on a nervous system, rendered more susceptible by the pregnant state. On the following day, Tuesday, she was unusually ill, suffered a number of the paroxysms mentioned, and drank a considerably large amount of alcoholic liquor. She was still worse the following day, Wednesday, but apparently not too ill, in her husband's opinion, to render him incompetent to prescribe for her, though a physician had visited her both Tuesday and Wednesday. On Thursday morning she was so much worse that a second physician was called, who reached her about noon, and found her so seriously ill as to give him great solicitude. He found her suffering nausea and frequent vomiting, insatiable thirst, and a sense of burning at the stomach, great restlessness, getting out of and into bed, muttering, and often noisy delirium, with hallucinations of sight and hearing, though she would obey orders, and reply to simple questions, apparently rationally.

Even up to a very few hours before her death, which took place some eighteen hours after the above visit, when spoken to she would respond, and so far as could be seen, rationally, but in a moment she would again relapse into wandering delirium. It also appears that, up to within a short time before her death, she was able to and did rise from her bed and walk about the room—a tendency her physicians say they tried to restrain, knowing that such efforts, in her exhausted condition, might suddenly destroy her life. There is no evidence I can find that she suffered any paroxysm to which the name of convulsion could be properly applied, during the last two or more days of her life.

A consultation, attended by three physicians, was had at eight o'clock on Thursday evening, and resulted in the conclusion that, among the complications of the case, there was *uræmia.*

We are not, however, informed if there were any dropsical swellings, œdema of the lungs, or if there had been any headache, or even if the urine had been examined by the physicians in attendance. On the contrary, they state distinctly that they did not examine it, but one of them gave some of the urine to an outside party, who testifies that he examined it and found *purpurine.* It is frankly acknowledged by the attending physicians that, up to the hour of the consultation, they had not arrived at a definite diagnosis of the case, and, even after the diagnosis agreed upon at the consultation, there seems to have been much doubt as to its correctness, for, either to ascertain a cause of death, or to corroborate an opinion formed before death, an autopsy was held. In the meager records of this *post-mortem* examination given us, nothing is said of any organ till the stomach is reached, and of this it is stated, that it was found somewhat congested, not much changed from health. It is said that the kidneys were both fatty and the liver extremely so. A dead fœtus was found in the womb, and one witness ventures so far as to give his opinion that it had been dead about forty-eight hours. This was of course a very hazardous opinion, if we are to judge it in the light of the fact that there does not occur a particle of evidence that the condition of the fœtus was even considered before the death of the mother. The only testimony I can find, which even alludes to the condition of the fœtus before the death of the mother, or to the question of parturient pains, is an equally hazardous statment of one medical witness to the effect that, if the fœtus had died three or four days before the mother's death, labor-pains would have come on earlier. Nothing is better known than the fact that a dead fœtus may and often does remain in the womb for long and indefinite periods without the occurrence of labor-pains. It does not appear in evidence that the alleged testatrix in this case had labor-pains at all. However, the conclusion from the autopsy was, that death resulted from uræmia, caused by Bright's disease of the kidneys, though no pathological record is offered in support of that *post-mortem* diagnosis, except the vague declaration that the kidneys were fatty, a condition often associated with fatty, light-colored livers in habitual drunkards, but not by any means necessarily accompanied with notable functional derangement of such kidneys.

On the other hand, the whole history of the symptoms of the case, as detailed in the evidence, is quite inconsistent with this eleventh-hour *post-mortem* diagnosis. The medical witnesses agree that the condition of the blood known as uræmia, caused by disease of the kidneys, often produces paroxysms, variable in character, sometimes maniacal convulsions, and sometimes, instead, a gradually deepening state of coma. But, to account for the extraordinary and unprecedented fact that in this case the alleged uræmic paroxysms did not induce coma and abolish all sane consciousness, notwithstanding the case terminated fatally, one of the principal medical witnesses employs the following notable language, applicable to the condition of the patient eight or nine hours before death: "It was concluded that she was suffering from uræmia, and it was a subject of remark that the case was exceptional, inasmuch as her mental operations were so clear; it is quite usual in a case of that kind that the contrary should be the case." As a support to this conclusion, one medical witness is reported as saying that he had seen many cases of Bright's disease of the kidneys, and that he had not found the mind generally affected. It is perhaps too much to expect of a judge, or of lawyers, that they should have deemed it important to ascertain if this witness had had a tangible and reliable record of a case of Bright's disease of the kidneys, causing uræmic paroxysms in a woman far advanced in pregnancy, and yet which did not, at eight hours before the fatal termination, affect her mind.

I challenge the medical world to produce the record of such a case. If we exclude a few cases in which the pregnant woman, not even suspecting her imminent danger, is suddenly seized with uræmic eclampsia and her life destroyed in a few hours, no case of uræmia terminates fatally in the pregnant woman without seriously affecting the intellect many hours before death; in other words, fatal cases of uræmia in pregnant women always affect, more or less profoundly, the mental power.

The history of this case, and the pathological history of uræmic *eclampsia gravidarum*, fail to furnish the slightest grounds for the opinion that death was caused by uræmia. Had we even the usual local or general œdema attending Bright's disease, or its fibrine cylinders, and fatty, degenerate renal epithelium in the urine—which we have not—the symptoms of this case, as set forth in the evidence, would be conclusive against the theory of fatal uræmia.

The evidence certainly shows that, from Tuesday to after twelve o'clock Thursday night of the same week, this woman's condition was one of nervous agitation, insomnia, and finally of busy, muttering, and talkative delirium, from which she could be momentarily aroused by speaking to her—a condition rarely or never seen after the development of fatal uræmic eclampsia, and certainly never within twelve hours of the time of death.

Due regard for the well-known intelligence of the physicians in attendance and consultation, forbids the conclusion that, even at the consultation on Thursday, nine hours before death, there were not very grave doubts as to how much of a part uræmia played in the production of the symptoms. Had there been no such doubts, the line of treatment, approved by the best authority, would have been (what it was not) purely obstetrical, and obstetrical counsel and means would have been resorted to, which was not the case, so far as the evidence goes. Now, as no such measures were taken, and, so far as the evidence goes, the uterus did not make the slightest spontaneous effort to expel the fœtus, it is simply fair to conclude that the diagnosis of uræmia did not take very deep hold on the physicians of the alleged testatrix before her death. Had they adopted that diagnosis, they could hardly expect to be excused for abandoning the patient undelivered—as they did—some five or six hours before her death. Then the practical failure of three intelligent physicians to decide that uræmic poisoning existed in this case during the fatal illness, as well as the symptoms attending the "spells" or "paroxysms" suffered during the last year of life, disproves the theory, which has only the support of a most imperfect and unscientific *post-mortem* examination. Like too many *post-mortem* conclusions where toxical agents have destroyed life, this examination, according to all the evidence, was totally fruitless of truthful results. It did not demonstrate the cause of death.

Having discussed the improbability, if not impossibility, that uræmic poisoning was the cause of death in the case, I will now inquire into the other possible causes of that event.

If the testimony is correct—and it is so abundant both on the part of her medical attendants and others, and so corroborative one of the other, as to be convincing—the attacks from which she suffered during most of her married life, called "spells of fainting," or "spasms," by the witnesses, are very accurately described in most essays on hysteria. The testimony does not inform us whether the alleged testatrix had these paroxysms or fits before she became pregnant. This, however, is not to be regarded as of importance, it being a well-known fact that it is common for women to suffer this and other forms of nervous attacks, for the first time, after becoming pregnant. There was, however, in this case an especial cause for these nervous attacks—a cause which fortunately is not very frequent among our young and recently married women. I allude to the excessive use of alcohol. It is a fact, well understood by persons conversant with the subject, that habitual inebriety is a fruitful cause of hysterical attacks in women who have no hysteria when sober. On this account alcoholic hysteria is regarded as distinct a form of this neurosis, as alcoholic epilepsy. There is, besides, very conclusive evidence in the case, that this woman had often, during her married life, suffered other results of the excessive use of alcohol than fits of hysteria. By his frequent experience with the attacks, or fits, or paroxysms, of alcoholic hysteria, if not delirium, suffered by his wife, it appears the husband had found out the value of laudanum. It is distinctly declared, by as respectable a witness as the case furnishes, that by order of the husband, she bought ten cents worth of laudanum for the deceased on Wednesday before her death. It is not proved that she did not take it, and the attending physician contradicts the husband's statement that it was ordered by the physician.

The apothecary who sold this laudanum says that the amount equals twelve doses of opium for the adult, or from twelve to eighteen grains. There is no reliable evidence to disprove that, whatever the physician may have ordered, she took, in addition, this twelve to eighteen grains of opium, and an indefinite amount of alcohol, during Wednesday and part of Thursday.

Now, if the husband had not regarded himself as skilled in the management of the wife's attacks, whatever they may have been—and nothing but experience or special education could have created that self-confidence—he must be regarded as careless, or daring, or criminal, for having assumed to administer a potent drug on his own responsibility; for one of the physicians in attendance swears that, on the morning after this laudanum was purchased, and probably given, he and a neighboring physician found the patient in so critical a condition as to induce them to seek counsel. Taking the most generous view of the facts, in behalf of the husband, we must suppose that his experience with laudanum in her accustomed attacks had given him so much confidence in its efficacy that he was unprepared to appreciate the graver symptoms of her final attack. He probably was unaware of the immediate danger; having seen her recover from so many spells, he presumed she would from this one.

This is the most liberal interpretation of his behavior. But returning to her symptoms, we find them enumerated as follows:

Much nervous and muscular agitation, restlessness, and insomnia; vomiting and insatiable thirst, with a burning pain at the stomach; hallucination of sight and of hearing; muttering and talkative delirium, calling the names of persons absent or dead, and yet replying promptly to questions; obeying the requests of the physicians, and in a moment forgetting them; and finally her ability to walk about the room till near the hour of her dissolution.

It would appear that few experienced medical men could mistake the significance of that array of symptoms, especially when occurring in a person of the admitted habits of this alleged testatrix. The following lines from the great master of clinical description, Dr. Thomas Watson, most aptly and fully hit the symptoms presented by this case:

"If you question the patient about his disease, he answers quite to the purpose; describes in an agitated manner his feelings, puts out his tongue, and does whatever you bid him; but immediately afterward he is wandering from the scenes around him to some others that exist only in his imagination. Generally his thoughts appear to be distressful and anxious; he is giving orders that relate to his business to persons who are absent, or he is devising plans to escape from some imaginary enemy; he fancies that rats, flies, mice, reptiles, or other objects, are running over his bed, or that strangers are in his room. He rises to look suspiciously about the room, or to leave his bed, but is readily induced to lie down again.

"Upon inquiring into the history of the patient, in a large majority of instances you will find that he has been an habitual drunkard, and very frequently that for some reason or other this habitual stimulus has been diminished or taken away. Sometimes, however, it comes on in those who are perpetually fuddled, even though they have not intermitted their usual indulgence in drink." —"*Practice of Medicine.*"

The burning pain at the stomach, vomiting, and thirst, which were so marked symptoms in this case, are often absent in alcoholic delirium. But as relates to the mental phenomena, Dr. Watson's general description of delirium tremens comes wonderfully near to meeting every symptom shown by this alleged testatrix during her last illness.

It will be difficult, if not impossible, to account for her symptoms upon any other supposition than that they resulted from alcoholic poisoning. If that cause be admitted, it is very manifest that she was quite *non compos mentis* for at least two days preceding her death. All her declarations, expressed wishes, as respected persons and business, after Wednesday morning, were in all probability mere ravings, and totally unreliable. Among those declarations should probably be placed, that her husband had injured her by striking her in the abdomen, causing a black-and-blue stain. Though two female witnesses testify to this declaration, and to having seen the bruised spot, it is not by any means improbable that they themselves asked her if the bruise had not been made by the husband, to which she answered, "Yes." Persons under this state of mental derangement need only the suggestion, to charge anybody with any act.

The husband denies that the declaration has any foundation, and he is, under the circumstances, by far the more reliable.

The cautious testimony of the physician, who saw her between eleven and twelve o'clock Thursday night, is in all probability the most favorable representation that could be made of her intellectual condition. He declares, under oath, that she was not, in his opinion, able to fix her mind long enough upon any one subject to dictate any article in writing.

It is in evidence, however, that she would reply to questions promptly, and apparently rationally, and would do, so far as she could, anything her physician or friends desired her to. We have seen that such a condition is very far from indicating rationality. In certain states of alcoholic delirium, responses to all manner of questions are obtained. Whether asked if they wish to die, or wish a drink, such persons are very liable to give the same reply —"Yes," or "No;" and the same if asked if they wish to see a priest or a physician. It may, hence, be readily comprehended how the subject of such a condition of mind may be brought to reply "yes," to any number of questions relative to the distribution of property by will; or how, if requested to sign a paper, he would at least make an effort to do so, though it might result in a complete bungle.

This power to respond, and this readiness to obey commands, are terminated sometimes very suddenly by the occurrence of death after some extra effort. In other cases, both the physical and mental powers fail gradually, and life does not become extinct till some hours after response and volition have failed. So far as the testimony instructs us, this impossibility of obeying requests, and of intelligent response, was reached in the case of this alleged testatrix about 12 o'clock on Thursday night, and she died about five hours after. It appears that, between 11 and 12 o'clock, having been asked if she would sign a will, she said, "Yes;" the arrangements were at once made, she was held in a reclining position, a pen was put into her hand, the place of signature pointed out, and she was requested to sign, which she did in a very imperfect manner. This having been accomplished, it appears in evidence that either she, or some other party, conceived the idea of adding a codicil. What conscious part she had to do with said codicil may be fairly inferred from the fact that, after responding to some half-dozen lines, of which it consists, she was too near dead to make it practicable to get her signature to it. She died, so far as can be gathered from the testimony, from four to five hours after this effort with the codicil, and died of asthenia. If it be admitted that she was suffering from alcoholic excesses, and that the cause of her death was even only in part of that character, not only the story of her symptoms, but the whole history of the influence of alcoholic poisoning on the intellect, and the accumulated clinical history of alcoholic delirium, go to confirm the opinion that this alleged testatrix had *no conscious appreciation of what she did or said for at least twenty, and probably seventy hours before death.*

If, however, we admit that there was a uræmic complication, or that uræmia was the chief cause of death, we encounter another unpleasant yet important fact in the case. On Wednesday, the day before the fatal symptoms came on, the ten cents' worth of laudanum was bought and probably administered, Now, it is a recognized fact that the administration of opiates in uræmia is dangerous practice in ever so skilled a hand; and hence, if that were her condition, the result of that medication, on the husband's own responsibility, may have been disastrous, and will now, of course, never be known. It is just to the husband, however, to say that the symptoms on the following day, as described by the witnesses, all contradict the theory that she suffered from uræmia, or that, if she so suffered, she took laudanum on Wednesday.

But it is possible, however unprecedented it may be, for uræmia to be manifested in a rare form in this respect.

There are two points in the case about which the medical testimony and the lay testimony are positive; one is, that the deceased was unable to finish her last will and testament; the other is, that she died of some blood-poisoning, either uræmic or alcoholic, or possibly choleric. The only known circumstance in which the intellect of a person, fatally poisoned with urea, is sound and disposing, a few hours before death, occurs, as I have before said, in those cases where uræmic convulsions come on with little antecedent disturbance, and destroy life suddenly. This was not the case with this alleged testatrix, and therefore the idea that she died of uræmia, without convulsions, yet was of sound mind five hours before death, is quite untenable. The same law applies to the choleric or bile poison. On the other hand, if we accept the theory that her vitality gave way under the destructive effects of alcohol, she had no intellect, in all reasonable probability, for a day or two before death. In either case, we are without the slightest reason that this alleged testatrix had even an approach to a sound and disposing mind and memory after eight o'clock on the Thursday in question.

As this case would lose much of its instructive and practical features if some of the legal history of it were omitted, we will occupy a little time upon it:

As has been stated, there is no evidence, except from the principal heir, that the alleged testatrix knew anything about the contents of the proposed will. There is no evidence that it was read to her at the time she made the blundering effort to sign it. On the contrary, it is stated that it was not read. The parties solely interested in it either dictated it or witnessed it, or both; the husband, the father-in-law, and the brother-in-law, doing all these offices, and were all associated in business. The unsuccessful attempt to add a codicil was a most trifling matter pecuniarily, the whole amount thereby diverted being but a few hundred dollars.

The court before whom the case was heard, decided that the decedent was, at the time she signed the paper, of sound and disposing mind, and gets over the fact that the parties solely benefited by it should have had all to do with its dictation, and even with witnessing it, in the following way: "However indelicate or impolitic it may have been for the proponent to have persons so nearly related to him, as the subscribing witnesses to the will of his wife, yet they were not disqualified, and I can find no reason in law why their testimony should be discredited, unless there should be other testimony and circumstances than have been presented in this case to weigh against their credibility."

Had the testatrix and proponents of this alleged will been, during the last day of her life, isolated from mankind, in the midst of some desert, or on some rock in the sea, there would be some reason for this attempt of the court to present an excuse for the fact that the parties witnessing it were closely related to the principal heir, and known to have been indirectly largely benefited by it; but when it is known that these witnesses affixed their signatures to it in the heart of a large city, and at an hour of the day when neighbors, notaries and lawyers are at their residences, and easily obtained, he will find it difficult to offer any laudable or legitimate reason for that "impolitic" irregularity.

It is often very useful to take different views of pictures as well as courts. The uncertainties of the law are thereby often shown to be quite as great as those said to be proverbial of medicine.

The same court which issued the above opinion, also, not very far from the same time, issued and subscribed to the following:

"The law is very zealous against straining after probate, when the preparation of a will is made by a party interested, to be executed by a person of doubtful capacity, and requires that, to support such probate, there must be *strong proof of intention outside of the interested party*, who claims to have acted under instructions.

"The court must take a cautious view in deciding questions of law and fact. It is an established principle that, where capacity is *doubtful at the time of execution*, there must be proof of instructions or of *reading over*. A man in a languid, torpid state may easily acquiesce in signing his name to a will set before him, more especially when he knows that there is something in the paper which he wishes to take effect; the presumption also is strong against an act done by the agency of the party benefited; the act is not actually defeated, as it was by the civil law, *provided the intention can be fairly deduced from other circumstances*. Though the court will not impute fraud, it will require *strong proof of intention*."

There is no positive or even circumstantial proof that there was any intention on the part of the alleged testatrix to give all her property to her husband. In addition to what has been said in relation to the signing of the alleged will, it may be interesting to further quote from this same court as to the mere signing of a will being an index of the state of soundness of the mind of a testator. In the same opinion, above quoted from, it is said:

"The power to affix a signature to a will, or even to sign checks, does not prove sanity, so far as the law requires, to perform such a solemn act as the making of a last will and testament. They may be merely automatic acts." Such unquestionably was the act of signing the alleged will in this case. The admitting of this will to probate was very clearly a "strain" in violation of all approved rulings, and of previous opinions of the same court.

Courts have the power to give opinions, and the people have a right to decide if those opinions are consistent with common sense and previous rulings, and physicians have an occasional right to say if those rulings are sustained by pathological facts.—*New York Medical Journal.*

